# Comprehensive examination of radiative electromagnetic flowing of nanofluids with viscous dissipation effect over a vertical accelerated plate

**DOI:** 10.1038/s41598-022-25097-2

**Published:** 2022-11-29

**Authors:** Shankar Goud Bejawada, Yanala Dharmendar Reddy, Wasim Jamshed, Usman, Siti Suzilliana Putri Mohamed Isa, Sayed M. El Din, Kamel Guedri, M. Israr Ur Rehman

**Affiliations:** 1 Department of Mathematics, JNTUH University College of Engineering Hyderabad, Kukatpally, 500085 Telangana India; 2 Department of Mathematics, Anurag University, Venkatapur, Hyderabad, 500 088 Telangana India; 3 Department of Mathematics, Capital University of Science and Technology, Islamabad, 44000 Pakistan; 4 Department of Computer Science, National University of Sciences and Technology, Balochistan Campus (NBC), Quetta, 87300 Pakistan; 5 Institute for Mathematical Research, Universiti Putra Malaysia, 43400 UPM Serdang, Selangor Darul Ehsan Malaysia; 6 Center of Research, Faculty of Engineering, Future University in Egypt, New Cairo, 11835 Egypt; 7 Mechanical Engineering Department, College of Engineering and Islamic Architecture, Umm Al-Qura University, P. O. Box 5555, Makkah, 21955 Saudi Arabia; 8 School of Mathematics and Statistics, Central South University, Changsha, 410083 China

**Keywords:** Mathematics and computing, Physics

## Abstract

This research aims to establish the MHD radiating convective nanofluid flow properties with the viscous dissipation across an exponentially accelerating vertical plate. As the plate accelerates, its temperature progressively increases. There are two separate types of water-based nanofluids that include copper ($Cu$) and titanium dioxide ($Ti{O}_{2}$) nanoparticles, respectively. The most crucial aspect of this investigation is finding a closed-form solution to a nonlinear coupled partial differential equations scheme. Galerkin finite element method (G-FEM) is used to figure out the initial managing equations. Utilizing graphs, the effect of the flow phenomenon's contributing variables as well as the influence of other factors is determined and depicted. In the part dedicated to the findings and discussion, the properties of these emergent parameters are described in more depth. Nonetheless, the thermal radiation and heat sink factors increase the thermal profile. In addition, the greater density of the copper nanoparticles cause the nanoparticle volume fraction to lessen the velocity delineation.

## Introduction

Heat transfer is the heat propagation between two separate structures or surroundings. Heat is propagated through conducting material (conduction), fluids (convection), and electromagnetic waves (radiation). The primary condition of heat transfer is the temperature of the structures and surroundings must be different (there must be a temperature gradient between these two systems or regions). Some of the applications of heat transfer in the industry are heat exchangers^1^, pulsed spray cooling^2^, magnetic cooling^3^, thermal reservoir^4^, etc.

Nanofluid is a colloidal solution that contains one type of particles in nanometer-sized in a based liquid, which is known as nanoparticles. The selected nanoparticles are oxides, metals, carbides, or carbon nanotubes, while the base fluids are water, oil, and ethylene glycol. The properties of the nanofluid are more significant than a conventional fluid which enhance the thermal conductivity, specific heat, and viscosity of the liquid. The potential usage of nanofluid has been applied in industrial cooling for tremendous energy savings and resulting in emissions reductions nanofluid coolant in automotive applications for smaller size and the more excellent location of the radiators^5^, computer cooling system^6^, etc. Tiwari and Das model treated the fluid, velocity, and temperature as constant, because this model is single phase^7^. The nanoparticles and base fluid elements in this model are assumed to be in thermal equilibrium, and the in-contact condition between these two elements is non-slip. In addition, the influence of nanoparticles volume fraction is also being deliberated. The implemented Tiwari-Das model has been reported recently, with the various two-dimensional model such as when the nanofluid is flowing over a stretching sheet^8^, porous media^9^, and cylinder^10^ etc.

Titanium dioxide (TiO_2_) is one type of nanoparticle with diameters less than 100 nm. Because of the bright whiteness owned by TiO_2_ and it is being considered safe, it is used in products such as food additives^11,12^. These two nanoparticles (Cu and TiO_2_) can be submerged simultaneously in the same base liquid for hybrid nanofluid preparation, where the hybrid nanofluid flow and thermal properties are affected by suction and viscous dissipation effects^13^, thermal radiation^14^, or porous medium^15^. Khan et al.^16^ investigated the Cu-TiO_2_ hybrid nanofluid, where its flow is induced by non-Fourier heat flux.

The boundary layer flow beyond a vertical plate or surface has been observed in many industrial processes, namely nuclear reactors, filtration procedures, drying porous materials in textile manufacturing, etc.^17^. The nanofluid flow beyond a semi-infinite plate due to the thermal conductivity is reported by Loganathan and Sangeetha^18^. The inclined magnetic field is included in the nanofluid flow model, which is bounded by convective conditions^19^. Haider et al.^20^ analyzed the unsteady state of hybrid nanofluids flow over an oscillating infinite vertical plate, with the effect of Newtonian heating.

Recently, the magnetic field in fluid convection (known as magnetohydrodynamics MHD) have enormous applications in medical science like thermo-chemotherapy^21,22^, On the other hand, thermal radiation acts as a heat transfer controller in polymer processing^23^, and solar power operation^24^. Moreover, the MHD radiating nanofluid flow over a horizontal plate or surface has been published for flat sheet^25^, cylinder^26^.

The Galerkin finite element technique provides numerical solutions^27^. Firstly, the multiplication of the weight function is performed to obtain the integral in the domain. Subsequently, together with the trail function, the step function is also selected with the order of interpolation. Then, each element was numerically calculated by integration to get the equations system. Finally, this system is solved to obtain the final solution. The hybrid nanofluid contains nanoparticles of copper and magnetite (Fe_3_O_4_), which flow over an infinite porous surface and is studied by Alkathiri et al.^28^ for thermal properties with the presence of entropy generation. The thermal performance of MHD radiating Williamson nanofluid flow bounded by infinite convective surface, with aluminum alloys (AA7072) and titanium alloy (Ti6Al4V) are studied by Hussain et al.^29^. They investigated the controlled factors of their mathematical model, known as viscous dissipation, Brownian, Joule heating, and thermophoresis diffusion. Pasha et al.^30^ studied the thermal radiation acting on the Powell-Eyring Cu-TiO_2_ hybrid nanofluid flow over an infinite slippery surface. The magnetohydrodynamics Ag-MgO hybrid nanofluid fills inside a porous triangular cavity were reported by Redouane et al.^31^. The flow and thermal features of Sutterby Cu-GO hybrid nanofluid over a slippery porous surface are investigated by Bouslimi et al.^32^, affected by viscous dissipation, thermal radiation, and solid-shaped nanoparticles. The mixed convection Maxwell MoS-Ag hybrid nanofluid over an infinite porous stretching sheet and the heat is generated or absorbed is considered by Algehyne et al.^33^. The Cattaneo–Christov heat flux model in the hybrid nanofluid flow over two distinct shapes^34^ and two parallel rotating disks^35^. Yaseen et al.^25,36–38^ have analyzed the following models of hybrid nanofluid flow: between two parallel Darcy porous plates^36^, over a extending or compressing wedge with the implementation of Falkner–Skan Problem^37^, over an irregular variably thick convex/concave‐shaped porous medium sheet^38^, and past a permeable moving surface with with assisting and opposing flow^25^. Priya et al.^39^ have inspected the radiating micropolar hybrid nanofluid flow past a vertical porous plate. Rawat and Kumar^40^ studied the copper water nanofluid with the utilization of Cattaneo–Christov heat flux model. The double‐diffusion copper–water nanofluid flow model with the employment of Cattaneo–Christov scheme and Stefan blowing has been discussed by Negi et al.^41^. The nanofluid flow over a vertical Riga plate is studied by Sawan et al.^42^.

In light of the previously mentioned information, this paper is desired to examine nanofluid optically thick radiative MHD free convection flow across the exponentially accelerating porous plate. In addition, free convection become the main factor since it is caused by the thermal buoyancy outcome, and the innovation of this paper is the incorporation of both heat sinks and thermal radiation in the energy equation. Many researchers have focused on the boundary layer flows of nanofluids induced by vertical plates. Their immense importance in engineering and industrial applications has been the driving force behind this development. These applications are especially prevalent in extrusion operations, the production of paper and glass fibre, the fabrication of electronic chips, the application of paint, the preparation of food, and the transfer of biological fluids. It is worthwhile to employ the Galerkin finite element technique, despite the fact that the differential equations are incomplete because of the instability. Various flow parameters' behaviors are obtained and explained graphically.

## Formulation of the problem

This work takes into consideration

**Figure 1:**
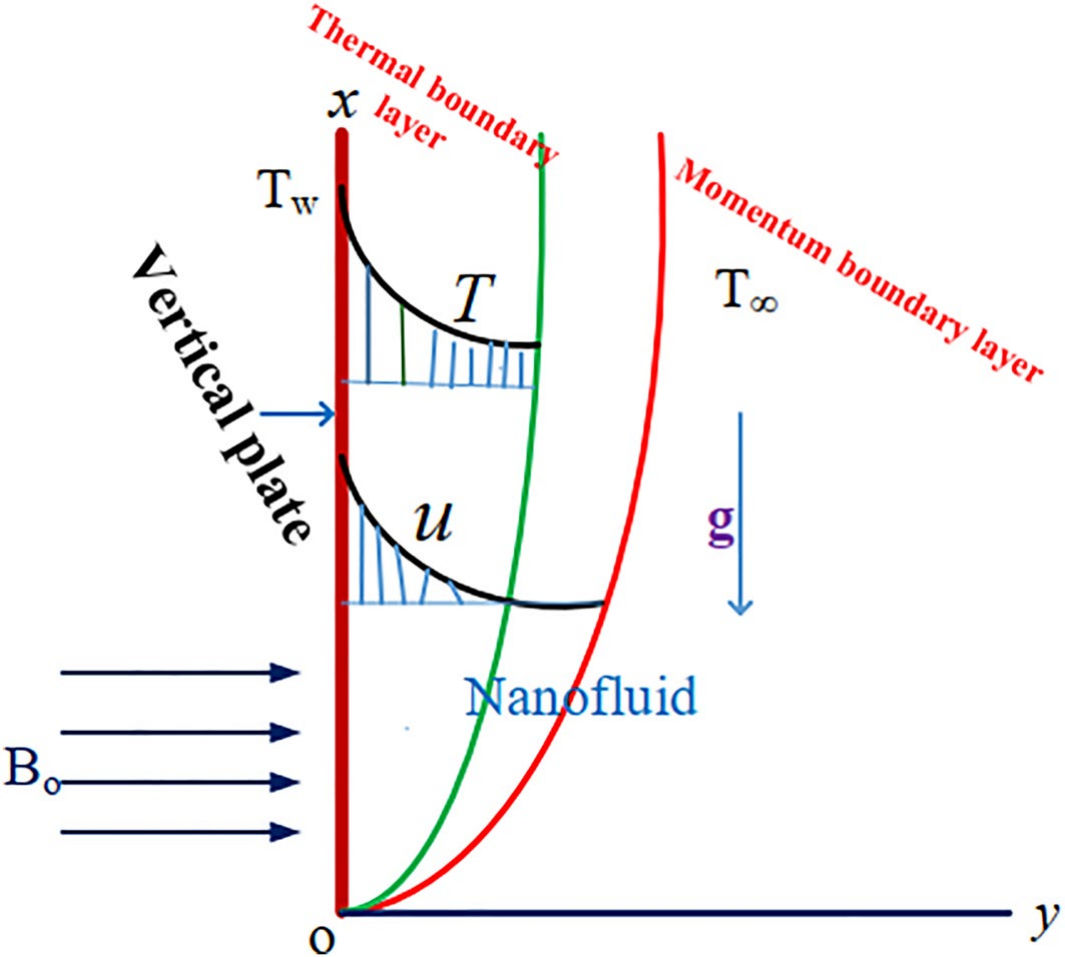
Geometrical flow of the problem.

1. optically thick water-based electrically conducting radiative MHD nanofluid flow along an accelerated exponentially ramping wall temperature integrated with permeable medium, positioned vertically upward.
2. It is well known that the flow occurs along the ${x}^{*}$ axis and that the ${y}^{*}$ axis represents its transverse direction.
3. For ${t}^{*}\le 0$, it is assumed that there is no motion in the fluid., i.e., no flow happens.
4. The plate velocity is supposed to be increased exponentially, i.e., ${U}_{0}{e}^{{a}^{*}{t}^{*}}$ along the flow direction, and the plate temperature of the plate is supposed to be unchanged, i.e., ${T}_{w}^{*}$.
5. A magnetic field (strength ${B}_{0}$) that intersects with the vertical plate, is provided to the flow in a normal direction (Fig. 1).

The governing equations for nanofluids, articulated in vector version, are as follows:

1 $$\nabla \cdot U = 0$$

2 $${\rho }_{nf}\frac{\partial U}{\partial t}=-{\nabla }_{p}+{\mu }_{nf}{\nabla }^{2}U+J\times B+F,$$

3 $$\left( {\rho c_{p} } \right)_{nf} \frac{\partial T}{{\partial t}} = - \nabla \cdot {\text{q}},$$

where $U=\left(u,v,w\right),q=-{k}_{nf}\nabla \mathrm{T}$ the heat flux, and $J={\sigma }_{nf}\left(E+U\times B\right)$ the current density.

Consideration is also given to the Rosseland-based radiative heat flow ${q}_{r}$. Considered here to be an isolated pressure gradient. Water containing nanoparticles of metals such as copper $(Cu)$ and Titanium oxide $Ti{O}_{2}$ is regarded as a nanofluid. The Eqs. (4–6) are inspired by Das and Jana^43^ and they are presented as below:

4 $${\rho }_{nf}\frac{\partial {u}^{*}}{\partial {t}^{*}}={\mu }_{nf}\frac{{\partial }^{2}{u}^{*}}{\partial {y}^{*2}}-{\sigma }_{nf}{B}_{0}^{2}{u}^{*}-\frac{{\mu }_{nf}}{K}{u}^{*}+g{\left(\rho {\beta }_{T}\right)}_{nf}({T}^{*}-{T}_{\infty }^{*})$$

5 $${(\rho {C}_{p})}_{nf}\frac{\partial {T}^{*}}{\partial {t}^{*}}={k}_{f}\frac{{\partial }^{2}{T}^{*}}{\partial {{y}^{*}}^{2}}-\frac{\partial {q}_{r}^{*}}{\partial {y}^{*}}+{Q}_{o}\left({T}^{*}-{T}_{\infty }^{*}\right)+{\mu }_{nf}{\left(\frac{\partial {u}^{8}}{\partial {y}^{*}}\right)}^{2}$$

6 $$\left.\begin{array}{c}t\le 0:{u}^{*}=0, {T}^{*}={T}_{\infty }^{*}, \quad \forall y\ge 0,\\ t>0:{u}^{*}={U}_{0}{e}^{{a}^{*}{t}^{*}}, {T}^{*}={T}_{w}^{*}, \quad at\, y=0,\\ t>0:{u}^{*}\to 0, {T}^{*}\to {T}_{\infty }^{*} \quad as\, y\to \infty \end{array}\right\}$$

Applying the Rosseland approximation at this point

7 $${q}_{r}=-\frac{4{\sigma }^{*}}{{k}^{*}}\frac{\partial {T}^{*4}}{\partial {y}^{*}}$$

When ${T}^{*4}$ is simplified further using Taylor's series expansion regarding ${T}_{\infty }^{*}$ and when just linear terms are considered, we get

8 $${T}^{*4}\approx 4{T}_{\infty }^{*3}{T}^{*}-{3T}_{\infty }^{*4}$$

Using the preceding expression, Eq. (6) has the following form:

9 $${\left(\rho {c}_{p}\right)}_{nf}\frac{\partial {T}^{*}}{\partial {t}^{*}}={k}_{nf}\frac{{\partial }^{2}{T}^{*}}{\partial {y}^{*2}}+\frac{16{T}_{\infty }^{*3}{\sigma }^{*}}{3{k}^{*}}\frac{{\partial }^{2}{T}^{*}}{\partial {y}^{*2}}+{Q}_{0}({T}^{*}-{T}_{\infty }^{*})+{\mu }_{nf}{\left(\frac{\partial {u}^{8}}{\partial {y}^{*}}\right)}^{2}$$

The following is a summary of the nanofluid's physical characteristics, based on Das and Jana's^43^ research:

$${\rho }_{nf}=\left(1-\phi \right){\rho }_{f}+\phi {\rho }_{s}, {\mu }_{nf}=\frac{{\mu }_{f}}{{\left(1-\phi \right)}^{2.5}},$$

$${\left(\rho {c}_{p}\right)}_{nf}=\frac{\left(1-\phi \right){\left(\rho {c}_{p}\right)}_{f}+\phi {\left(\rho {c}_{p}\right)}_{s}}{\left(\rho {c}_{p}\right)},$$

$${\left(\rho {\beta }_{t}\right)}_{nf}=\frac{\left(1-\phi \right){\left(\rho {\beta }_{t}\right)}_{f}+\phi {\left(\rho {\beta }_{t}\right)}_{s}}{\left(\rho {\beta }_{t}\right)},$$

$${\sigma }_{nf}={\sigma }_{f}\left[1+\frac{3\left(\frac{{\sigma }_{s}}{{\sigma }_{f}} -1\right)\phi }{\left(\frac{{\sigma }_{s}}{{\sigma }_{f}}+2\right)-\left(\frac{{\sigma }_{s}}{{\sigma }_{f}} -1\right)\phi }\right],$$

$${k}_{nf}={k}_{f}\left\{\frac{{k}_{s}+2{k}_{f}-2\phi ({k}_{f}-{k}_{s})}{{k}_{s}+2{k}_{f}+\phi ({k}_{f}-{k}_{s})}\right\}$$

With the aid of the following dimensionsless variables

$$y=\frac{{y}^{*}}{{U}_{0}{t}_{0}},t=\frac{{t}^{*}}{{t}_{0}},u=\frac{{u}^{*}}{{U}_{0}},T=\frac{{T}^{*}-{T}_{\infty }^{*}}{{T}_{w}^{*}-{T}_{\infty }^{*}}$$

And substituting in Eqs. (4), (6), and (9), we get:

10 $$\frac{\partial u}{\partial t}={r}_{1}\frac{{\partial }^{2}u}{\partial {y}^{2}}+{r}_{2}Gr\theta -\left({r}_{3}M+\frac{1}{{K}_{p}}\right)u,$$

11 $$\frac{\partial \theta }{\partial t}={r}_{4}\frac{{\partial }^{2}\theta }{\partial {\eta }^{2}}+{r}_{5}\theta +{r}_{6}Ec{\left(\frac{\partial u}{\partial \eta }\right)}^{2}$$

Surface conditions are

12 $$\left.\begin{array}{c}t\le 0:u=0, \theta =0,\quad \forall y\ge 0,\\ t>0:\left\{\begin{array}{c}u={e}^{at}, \theta =1, \quad at\, y\ge 0\\ u\to 0,\theta \to 0\quad as \,y\to \infty \end{array}\right.\end{array}\right\}$$

where $M=\frac{{\sigma }_{f}B{z}_{o}^{2}{\nu }_{f}}{{U}_{0}{\rho }_{f}}$ is the magnetic field, $Gr=\frac{g{\beta }_{Tf}\left({T}_{w}-{T}_{\infty }\right){\nu }_{f}}{{U}_{0}^{3}}$ is the Modified Grashof number, $Pr=\frac{{\left(\mu {c}_{p}\right)}_{f}}{{k}_{f}}$ referees the Prandtl number, $R=\frac{4{\sigma }^{*}{T}_{\infty }^{3}}{{k}^{*}{k}_{f}}$ indicates the radiation parameter, $Q=\frac{Q{\nu }_{f}}{{U}_{0}^{2}{\left(\rho {c}_{p}\right)}_{f}}$ is the heat source/sink parameter and ${K}_{p}=\frac{K{\nu }_{f}}{{U}_{0}^{2}}$ is the porosity factor.

$$\begin{gathered} r_{1} = \frac{1}{{\left( {1 - \phi } \right)^{2.5} \left[ {\left( {1 - \phi } \right)\rho_{f} + \phi \frac{{\rho_{s} }}{{\rho_{f} }}} \right]}},\quad r_{2} = \frac{{\left( {1 - \phi } \right) + \phi \frac{{\left( {\rho \beta_{T} } \right)_{s} }}{{\left( {\rho \beta_{T} } \right)_{f} }}}}{{\left( {1 - \phi } \right)\rho_{f} + \phi \frac{{\rho_{s} }}{{\rho_{f} }}}},\quad r_{3} = \frac{{\left[ {1 + \frac{{3\left( {\frac{{\sigma_{s} }}{{\sigma_{f} }} - 1} \right)\phi }}{{\left( {\frac{{\sigma_{s} }}{{\sigma_{f} }} + 2} \right) - \left( {\frac{{\sigma_{s} }}{{\sigma_{f} }} - 1} \right)\phi }}} \right]}}{{\left( {1 - \phi } \right)\rho_{f} + \phi \frac{{\rho_{s} }}{{\rho_{f} }}}}, \hfill \\ r_{4} = \frac{1}{{\left( {1 - \phi } \right) + \phi \frac{{\left( {\rho c_{p} } \right)_{s} }}{{\left( {\rho c_{p} } \right)_{f} }}}} .\frac{1}{Pr} \left\{ {\frac{3}{4}R + \left\{ {\frac{{k_{s} + 2k_{f} - 2k\phi \left( {k_{f} - k_{s} } \right)}}{{k_{s} + 2k_{f} + \phi \left( {k_{f} - k_{s} } \right)}} } \right\}} \right\},\;\quad r_{5} = \frac{Q}{{\left( {1 - \phi } \right) + \phi \frac{{\left( {\rho c_{p} } \right)_{s} }}{{\left( {\rho c_{p} } \right)_{f} }}}},\quad r_{6} = \frac{1}{{\left( {1 - \phi } \right)^{2.5} x_{4} }} \hfill \\ \end{gathered}$$

The quantifiable thermal aspects of liquid and nanoparticles are presented in Table 1.

**Table 1:** Thermal features of liquid and nanoparticles at 293 K.

Thermophysical	*ρ* (kg/m)	*C*_*p*_ (J/kg K)	*κ* (W/mK)	β × 10^−5^ (K^−1^)	*σ* (S/m)
Water (H_2_O)	997.1	4179	0.613	21	5.5 $\times {10}^{-6}$
Copper (Cu)	8933	385	401	–	35 $\times {10}^{-6}$
Titanium oxide (TiO_2_)	4250	6862	8.9583	0.9	2.6 $\times {10}^{-6}$

## Problem solution: Galerkin finite element method

The Galerkin weighted residual numerical approach is implemented in conjunction with a robust FEM solution to deal with the dimensionless complex partial differential Eqs. (10–11) and (13). Following are the five stages that make up this full procedure.

Some of these steps involve.

### Step-1: discretization

During this step, the whole problem area is broken up into smaller parts called "finite elements." The component $\left(e\right)$ is expanded by using the Galerkin finite element technique for Eq. (10), is

13 $$\underset{{y}_{j}}{\overset{{y}_{k}}{\int }}\left\{{N}^{(e)}\left[{r}_{1}\frac{{\partial }^{2}{u}^{(e)}}{\partial {y}^{2}}-\frac{\partial {u}^{\left(e\right)}}{\partial t}+{r}_{2}Gr\theta -\left({r}_{3}M+\frac{1}{{K}_{p}}\right){u}^{\left(e\right)}+P\right]\right\}dy=0$$

Using the by-parts method to put the first part together

14 $${N}^{\left(e\right)}{\left\{\frac{\partial {u}^{(e)}}{\partial y}\right\}}_{{y}_{j}}^{{y}_{k}}-\underset{{y}_{j}}{\overset{{y}_{k}}{\int }}\left\{{N}^{(e)}\left[{r}_{1}\frac{\partial {N}^{(e)}}{\partial y}\frac{\partial {u}^{(e)}}{\partial y}+\frac{\partial {u}^{(e)}}{\partial t}-{r}_{2}Gr\theta +\left({r}_{3}M+\frac{1}{{K}_{p}}\right){u}^{\left(e\right)}-P\right]\right\}dy=0$$

Leaving out the first part of Eq. (14), the following can be found:

15 $$\underset{{y}_{j}}{\overset{{y}_{k}}{\int }}\left\{{N}^{(e)}\left[{r}_{1}\frac{\partial {N}^{(e)}}{\partial y}\frac{\partial {u}^{(e)}}{\partial y}+\frac{\partial {u}^{(e)}}{\partial t}-{r}_{2}Gr\theta +\left({r}_{3}M+\frac{1}{{K}_{p}}\right){u}^{(e)}\right]-P\right\}dy=0$$

### Step-2: derivation of the element equation

In this step by taking the linear solution to the component $y\in [{y}_{j},{y}_{k}]$ and the basis functions which are in this stage, the linear solution to the component $y\in [{y}_{j},{y}_{k}]$ and the basis functions, which are, are taken into consideration.

$${u}^{(e)}={N}^{(e)}{\psi }^{(e)}$$, here $${N}^{(e)}=\left[{N}_{j}, {N}_{k}\right], {\psi }^{e}={\left[{u}_{j}, {u}_{k}\right]}^{T}$$ and $${N}_{j}=\frac{{(y}_{k}-y)}{{(y}_{k}-{y}_{j})}, {N}_{k}=\frac{y-{y}_{k}}{{y}_{k}-{y}_{j})}$$

Incorporating into Eq. (15),

$$\underset{{y}_{j}}{\overset{{y}_{k}}{\int }}\left\{{r}_{1}\left[\begin{array}{cc}{N}_{j}^{^{\prime}}{N}_{j}^{^{\prime}}& {N}_{j}^{^{\prime}}{N}_{k}^{^{\prime}}\\ {N}_{j}^{^{\prime}}{N}_{k}^{^{\prime}}& {N}_{k}^{^{\prime}}{N}_{k}^{^{\prime}}\end{array}\right]\left[\begin{array}{c}{u}_{j}\\ {u}_{k}\end{array}\right]+\left[\begin{array}{cc}{N}_{j} {N}_{j}& {N}_{j} {N}_{k}\\ {N}_{j} {N}_{k}& {N}_{k} {N}_{k}\end{array}\right]\left[\begin{array}{c}\dot{{u}_{j}}\\ \dot{{u}_{k}}\end{array}\right]+\left({r}_{3}M+\frac{1}{{K}_{p}}\right)\left[\begin{array}{cc}{N}_{j} {N}_{j}& {N}_{j} {N}_{k}\\ {N}_{k} {N}_{j}& {N}_{k} {N}_{k}\end{array}\right]\left[\begin{array}{c}{u}_{j}\\ {u}_{k}\end{array}\right]\right\}dy=P\left[\begin{array}{c}{u}_{j}\\ {u}_{k}\end{array}\right]$$

By reducing the above equation, we get:

16 $$\frac{{r}_{1}}{{l}^{e}}\left[\begin{array}{cc}1& -1\\ -1& 1\end{array}\right]\left[\begin{array}{c}{u}_{j}\\ {u}_{k}\end{array}\right]+\frac{{l}^{e}}{6}\left[\begin{array}{cc}2& 1\\ 1& 2\end{array}\right]\left[\begin{array}{c}\dot{{u}_{j}}\\ \dot{{u}_{k}}\end{array}\right]+\frac{\left({r}_{3}M+\frac{1}{{K}_{p}}\right)}{2}\left[\begin{array}{cc}-1& 1\\ -1& 1\end{array}\right]\left[\begin{array}{c}{u}_{j}\\ {u}_{k}\end{array}\right]=P.$$

### Step-3: assemble the element equations

The following may be accomplished by assembling the element equations for consecutive components ${y}_{i-1}\le y\le {y}_{i}$ and ${y}_{i}\le y\le {y}_{i+1}$ in the following stages

17 $$\frac{{r}_{1}}{{l}^{{\left(e\right)}^{2}}}\left[\begin{array}{ccc}1& -1& 0\\ -1& 2& -1\\ 0& -1& 1\end{array}\right]\left[\begin{array}{c}{u}_{i-1}\\ {u}_{i}\\ {u}_{i+1}\end{array}\right]+\frac{1}{6}\left[\begin{array}{ccc}2& 1& 0\\ 1& 4& 1\\ 0& 1& 2\end{array}\right]\left[\begin{array}{c}\dot{{u}_{i-1}}\\ \dot{{u}_{i}}\\ \dot{{u}_{i+1}}\end{array}\right]+\frac{\left({r}_{3}M+\frac{1}{{K}_{p}}\right)}{2{l}^{e}}\left[\begin{array}{ccc}-1& 1& 0\\ -1& 0& 1\\ 0& -1& 1\end{array}\right]\left[\begin{array}{c}{u}_{i-1}\\ {u}_{i}\\ {u}_{i+1}\end{array}\right]=P$$

After setting ‘*i*’ to 0 in the specified node row, the change pattern with "${l}^{e}$=h" in Eq. (17) is

$$\frac{1}{6}\left[\dot{{u}_{i-1}}+4\dot{{u}_{i}}+\dot{{u}_{i+1}}\right]+\frac{{r}_{1}}{{l}^{{\left(e\right)}^{2}}}\left[-{u}_{i-1}+2{u}_{i}-{u}_{i+1}\right]+\frac{\left({r}_{3}M+\frac{1}{{K}_{p}}\right)}{2{l}^{e}}\left[-{u}_{i-1}+{u}_{i+1}\right]={P}^{*}$$

The utilization of trapezoidal rule produces the Crank–Nicholson equations systems:

18 $${A}_{1}{u}_{i-1}^{n+1}+{A}_{2}{u}_{i}^{n+1}+{A}_{3}{u}_{i+1}^{n+1}={A}_{4}{u}_{i-1}^{n}+{A}_{5}{u}_{i}^{n}+{A}_{6}{u}_{i+1}^{n}+{P}^{*}$$

where $$A1=\left(2\right)-\left(6*r*r1\right)+\left(k*\left({r}_{3}M+\frac{1}{{K}_{p}}\right)\right);\,\, A2=\left(8\right)+\left(12*r*r1\right)+\left(4*k*\left({r}_{3}M+\frac{1}{{K}_{p}}\right)\right); \,\, A3=\left(2\right)-\left(6*r*r1\right)+\left(k*\left({r}_{3}M+\frac{1}{{K}_{p}}\right)\right); \,\, A4=\left(2\right)+\left(6*r*r1\right)-\left(k*\left({r}_{3}M+\frac{1}{{K}_{p}}\right)\right); \,\, A5=\left(8\right)-\left(12*r*r1\right)-\left(4*k*\left({r}_{3}M+\frac{1}{{K}_{p}}\right)\right); \,\, A6=\left(2\right)+\left(6*r*r1\right)-\left(k*\left({r}_{3}M+\frac{1}{{K}_{p}}\right)\right); \,\, {P}^{*}=12{r}_{2}Gr\theta k$$

The same process is applied to the Eq. (11) obtained

19 $$G_{1} u_{i - 1}^{n + 1} + G_{2} u_{i}^{n + 1} + G_{3} u_{i + 1}^{n + 1} = G_{4} u_{i - 1}^{n} + G_{5} u_{i}^{n} + G_{6} u_{i + 1}^{n}$$

$$\begin{gathered} G1 = \left( 2 \right) - \left( {6*r*r4} \right) - \left( {k*r5} \right); G2 = \left( 8 \right) + \left( {12*r*r4} \right) - \left( {4*k*r5} \right); G3 = \left( 2 \right) - \left( {6*r*r4} \right) - \left( {k*r5} \right); \hfill \\ G4 = \left( 2 \right) + \left( {6*r*r4} \right) + \left( {k*r5} \right); G5 = \left( 8 \right) - \left( {12*r*r4} \right) + \left( {4*k*r5} \right); G6 = \left( 2 \right) + \left( {6*r*r4} \right) + \left( {k*r5} \right); \hfill \\ \end{gathered}$$

Here the $y$ and time direction of $h$ and $k$ are the mesh dimensions. $i, n$ indicate that the space and time, respectively.

Here, h and k represent mesh dimensions in the y and time directions, respectively. Where $i,$ and n represent space and time, appropriately.

### Step: imposing the boundary constraints

The set of equations is obtained from the boundary restrictions (12) in Eqs. (17, 18) where ${A}_{i}{X}_{i}={B}_{i}$ stands in place of for $i(i)=3$, ${A}_{i} , {X}_{i}$ and ${B}_{i}$ as matrices. Applying the Thomas algorithm with ${10}^{-6}$ accuracy via MATLAB-code execution yields the necessary numerical solutions.

The wall shear stress, t, and the thermal transmission rate are of great relevance in many technological contexts.

Skin friction (also known as shear stress) at the wall can be determined by:

20 $$\tau ={\left.\frac{\partial u}{\partial \xi }\right|}_{\xi =0}$$

The heat transmission coefficient at the wall, expressed as a Nusselt number (Nu) using the following formula

21 $$Nu = \left. {\frac{\partial \theta }{{\partial \xi }}} \right|_{\xi = 0} .$$

## Outcomes and analysis

The managing parameters like heat generation/absorption $Q$, magnetic $M$, radiation $R$, volume fraction $\phi$, porosity $K$, Grashoff number $Gr$, coefficient of exponent $a$, Eckert number $Ec$, and time $t$ for two nanoparticles $Cu-Water$ and ${TiO}_{2}-Water$ upon the non-dimensional distributions of velocity $u\left(\xi \right)$ and temperature $\theta \left(\xi \right)$ are examined along with $\xi$. The smooth lines are plotted to measure the effects of the $Cu-Water$ nanoparticle, whereas the dotted lines for ${TiO}_{2}-Water$ nanoparticle.

**Figure 2:**
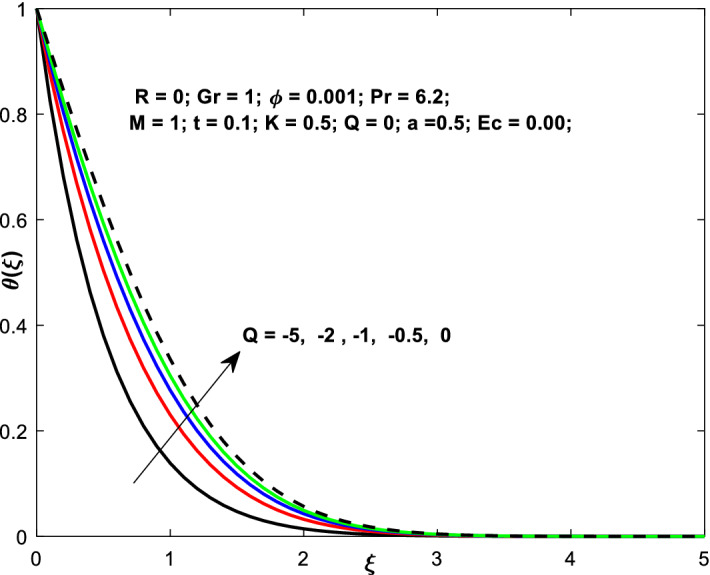
Q v/s $$\theta$$ when $$R=Q=Ec=0$$.

**Figure 3:**
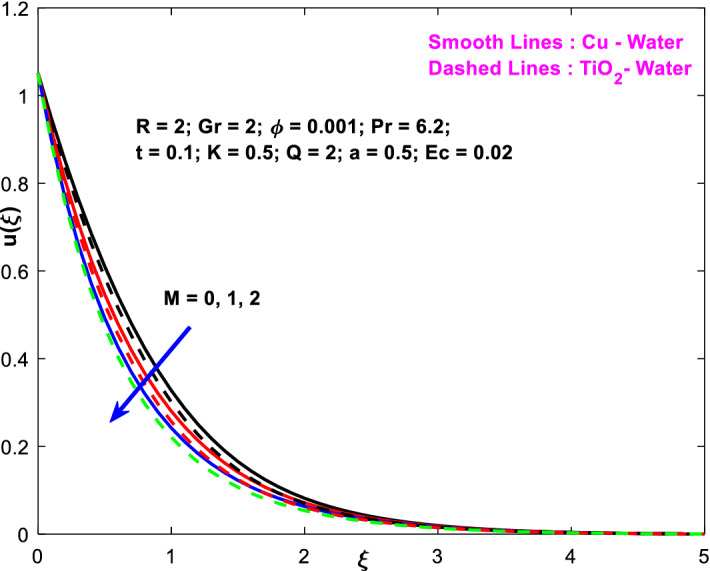
$$M$$ v/s $$u$$.

**Figure 4:**
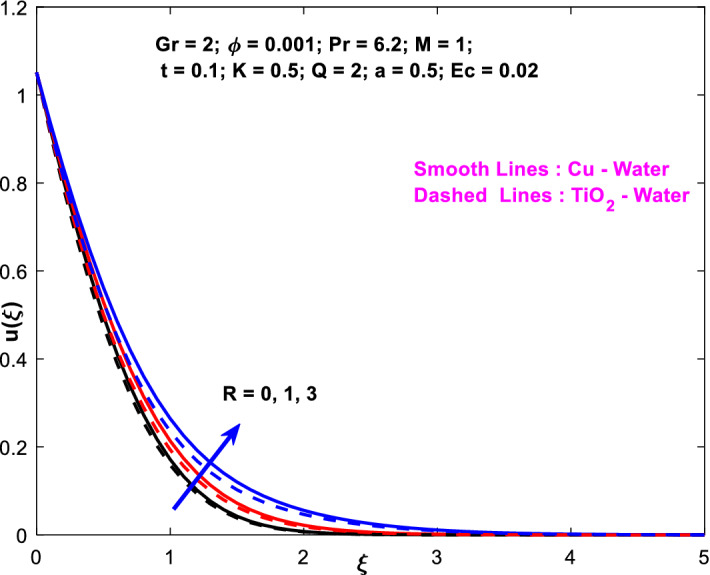
$$R$$ v/s $$u$$.

**Figure 5:**
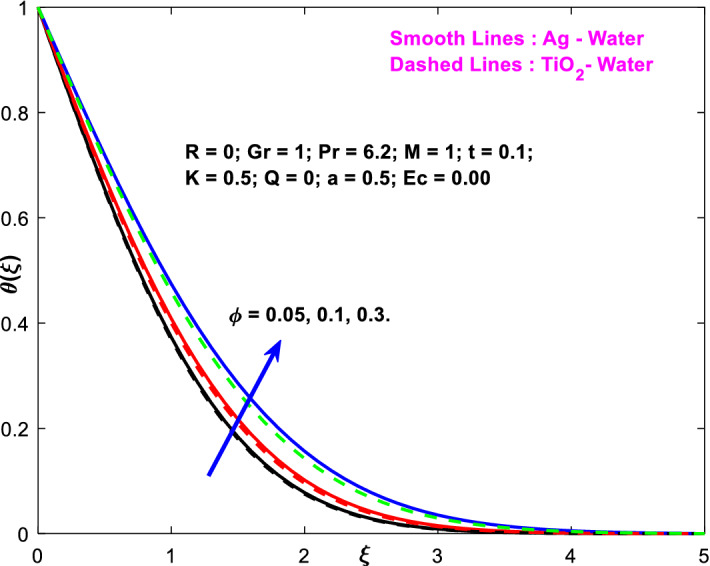
$$\phi$$ v/s $$\theta$$.

**Figure 6:**
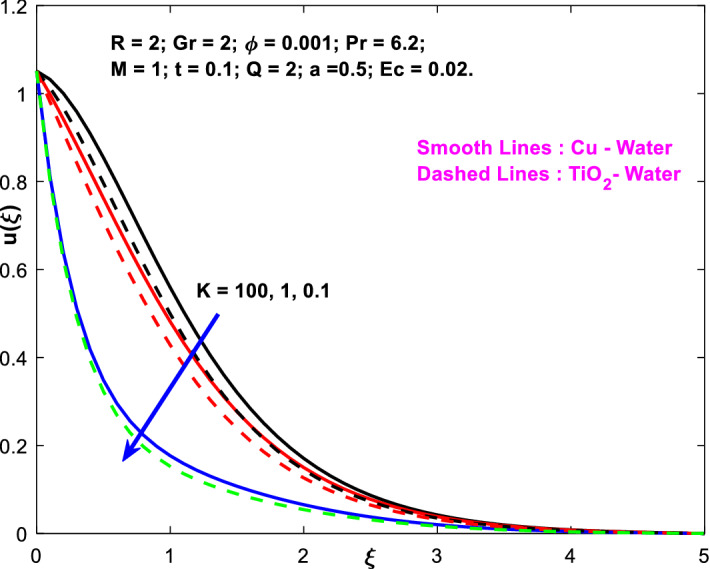
$$K$$ v/s $$u$$.

**Figure 7:**
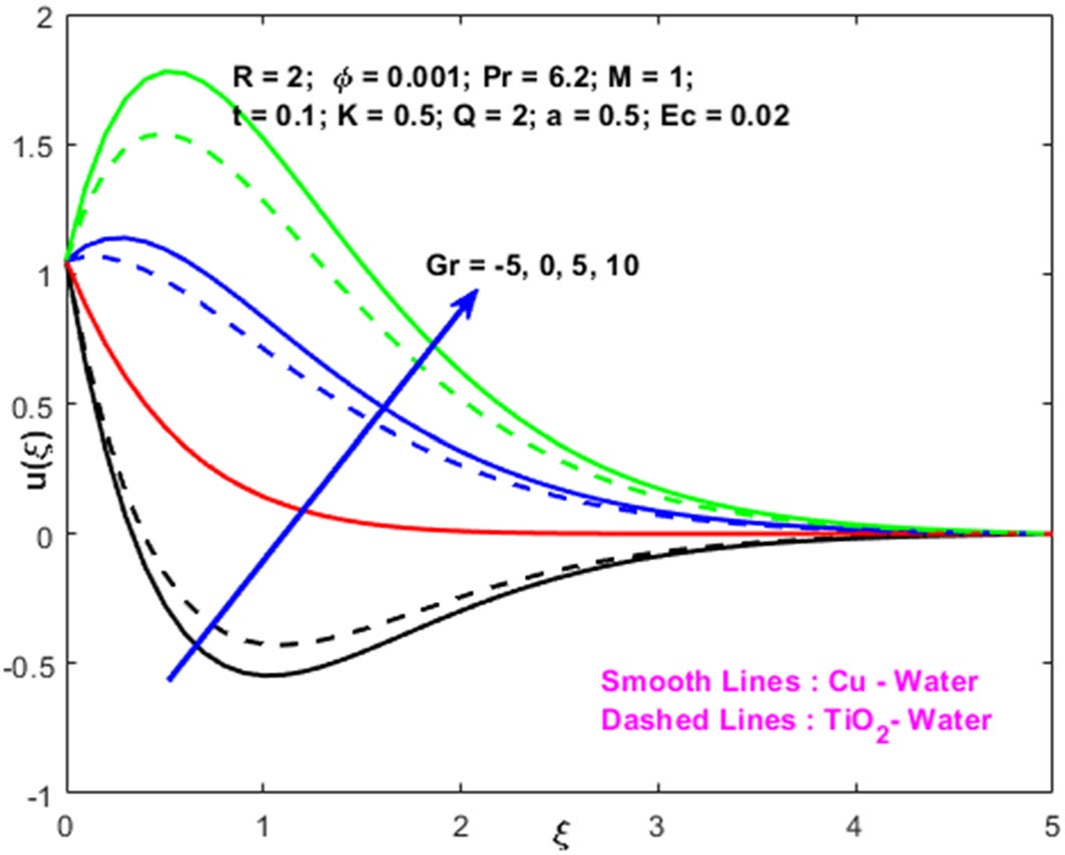
$$Gr$$ v/s $$u$$.

**Figure 8:**
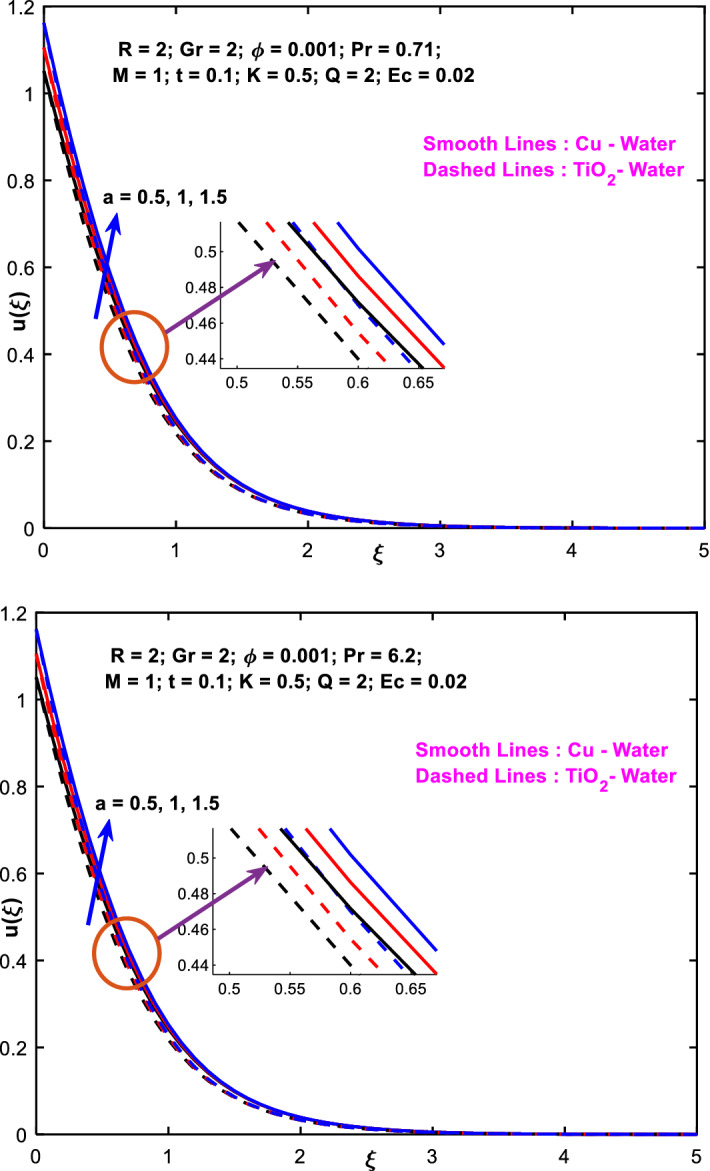
$$a$$ v/s $$u$$.

**Figure 9:**
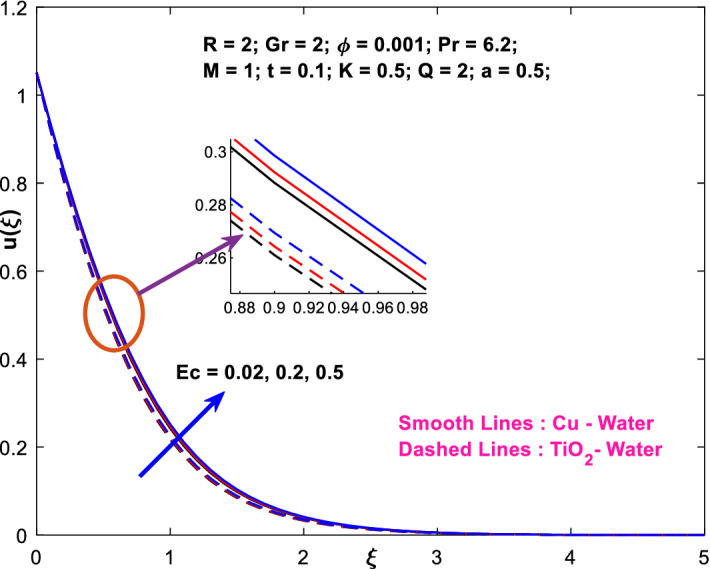
$$Ec$$ v/s $$u$$.

**Figure 10:**
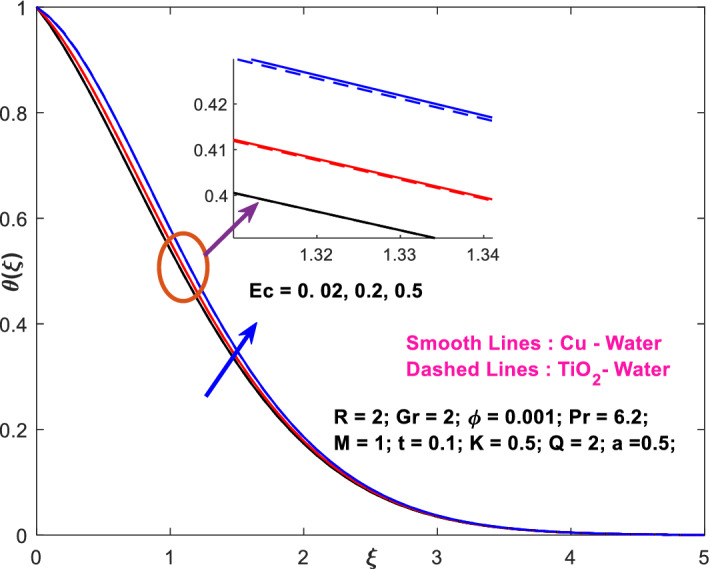
$$Ec$$ v/s $$\theta$$.

**Figure 11:**
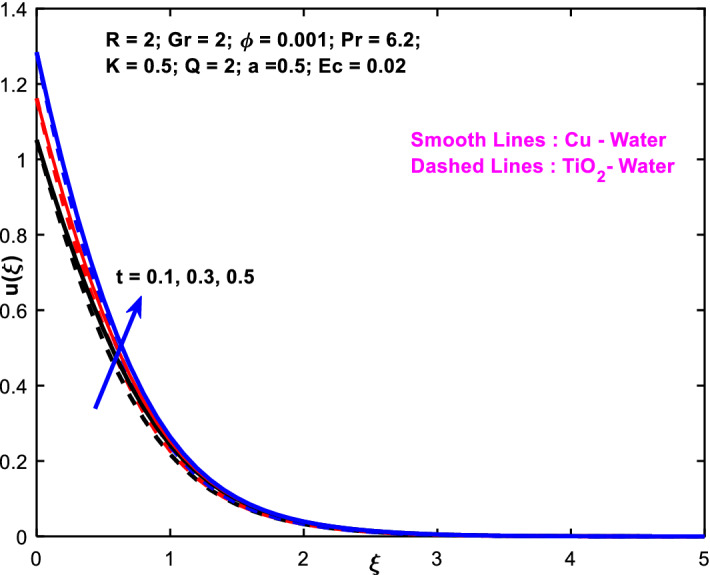
$$t$$ v/s $$u$$.

**Figure 12:**
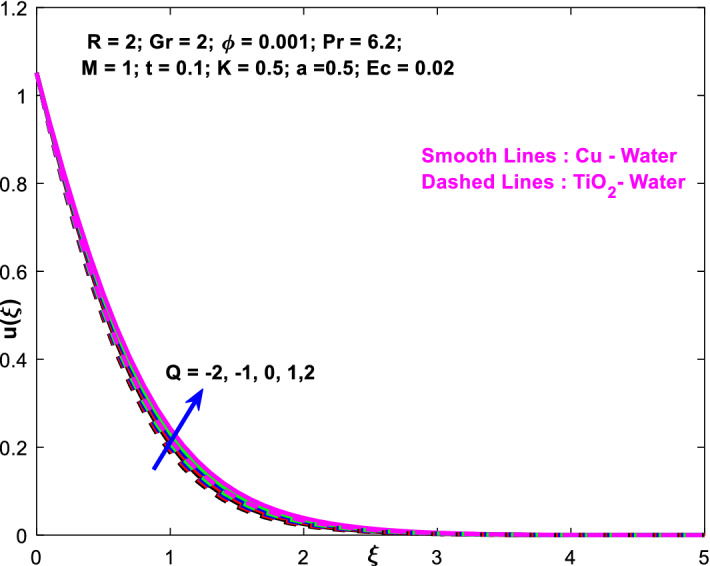
$$Q$$ v/s $$u$$.

**Figure 13:**
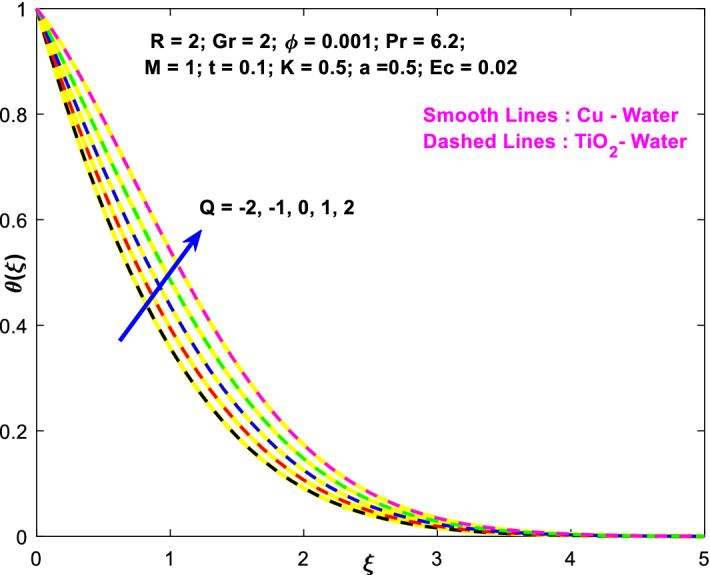
$$Q$$ v/s $$\theta$$.

The effects of the heat absorption $Q<0$ on the non-dimensional temperature $\theta \left(\xi \right)$ profile are delineated in Fig. 2 when $R=Ec=0$. It is depicted in Fig. 2 that when $Q=0,$ the profile has obtained its maximum value and gradually decreases when $Q<0$. Basically, $Q<0$ behaves like a heat sink; therefore, escalating $Q<0$ causes a deduction in the temperature due to the energy absorption during the heat sink process. In the same manner, a rising of fluid temperature causes a flow toward the plate as a result of the thermal buoyancy forces. Since the thickness of the momentum boundary layer is decreasing, the velocity is also decreasing as a result. It has also been seen that the velocity rises with the flow of time. The impression of $M$ upon $u\left(\xi \right)$ for $Cu-Water$ and ${TiO}_{2}-Water$ are portrayed in Fig. 3. This figure shows that the velocity profile has achieved its highest value in the non-existence of a magnetic region, i.e., when $M=0$. Besides, the profile decays when $M\ne 0$. The escalation in the parameter $M$ leads to the existence of the Lorentzian force, which shows a retarding behavior against the flow behavior. So, the Lorentz force opposes the fluid motion which consequently decreases the boundary layer thickness and velocity distribution. Also, the increase in magnetic parameter upsurges the frictional forces between the particles of the fluids. That’s why the velocity distribution is lower for higher magnetic factor. The decrease for ${TiO}_{2}-Water$ is slightly higher. Figure 4 is captured to predict the impression of $R$ on $u\left(\xi \right)$ for both nanofluid particles $\left(Cu-Water, {TiO}_{2}-Water\right)$. It is demonstrated from Fig. that the fluid velocity rises for increasing $R$. The rise for $Cu-Water$ is more extensive. Figure 5 is depicted the impression of $\phi$ upon $\theta \left(\xi \right)$ profile for $Cu-Water$ and ${TiO}_{2}-Water$. It is examined from Fig. 5 that the increment in $\phi$ leads to an escalation in $\theta \left(\xi \right),$ and this escalation is a little more extensive for $Cu-Water$. The cause of this behavior is that intermolecular interactions between the nanoparticles weaken with escalating the parameter $\phi$. Subsequently, the escalation of the thermal boundary layer thickness takes effect. Due to this fact, the temperature grows. Figure 6 measures the impact of $K$ on $u\left(\xi \right)$ for both nanofluid particles $\left(Cu-Water, {TiO}_{2}-Water\right)$. The fluid velocity decays as the parameter $K$ grows. It is described physically as the regime becoming more porous as the parameter $K$ increases. The Darcian force's strength decreases in this manner, slowing the mobility of the fluid's molecule particles. Consequently, the decrement of fluid velocity appears. The decrease for ${TiO}_{2}-Water$ is slightly more than from $Cu-Water$. The impact of Grashoff’s number $Gr$ upon a non-dimensional velocity profile $u\left(\xi \right)$ is measured for $Cu-Water, {TiO}_{2}-Water$. The profile is experienced increasing along $\xi$ for higher estimations of the parameter $Gr$. Where $Gr>0$ means the cool surface of the plate moreover $Gr<0$ signifies the hot surface of the plate. This is obvious from Fig. 7 that the cool surface increases the fluid velocity, whereas the hot surface decreases it. It is further seen that when $Gr<0$ the decrease for $Cu-Water$ is higher but when $Gr>0$ the increase for $Cu-Water$ is larger than from ${TiO}_{2}-Water$. The impact of $a$ on $u\left(\xi \right)$ for $Cu-Water, {TiO}_{2}-Water$ is depicted in Fig. 8. The profile $u\left(\xi \right)$ grows for higher estimations of the parameter $a$. From this description, From this description, obvious to say from the definition that as the parameter $a$ escalates the characteristic of the exponential function too escalates quickly. This can be observed from Fig. that when the parameter $a$ rises the velocity profile also rises within the domain for $Cu-Water, {TiO}_{2}-Water$. This rise is marginally greater for $Cu-Water$. The outcome of $Ec$ upon $u\left(\xi \right)$ and $\theta \left(\xi \right)$ outlines are summarized in Figs. 9 and 10 both nanofluid particles $\left(Cu-Water, {TiO}_{2}-Water\right)$. It is experienced from these Figs. 9 and 10. That both profiles escalate for growing estimations of the parameter $Ec$. The viscous dissipation impact is predicted by the Eckert number *Ec*. As the $Ec$ grows the kinetic energy is converted into heat energy. Consequently, the thermal conductivity is enhanced and the fluid temperature elevates. The fluid velocity and temperature are marginally higher for $Cu-Water$ than from ${TiO}_{2}-Water$ when the parameter $Ec$ rises. Figures 11, 12 and 13 are plotted to elucidate the impacts of $t$ and $Q$ of $u\left(\xi \right)$ and $\theta \left(\xi \right)$ outlines for $Cu-Water, {TiO}_{2}-Water$. It is examined from Figs. 11, 12, 13 that the velocity and temperature increase for the parameters $t$ and $Q$ (see Figs. 11, 12). If $Q<0$ then this means the absorption process and behaves like a heat sink which reduces the velocity and the temperature. Whereas, if $Q>0$ then this leads to a generation process and it acts like a heat source that increases velocity and temperature.

The numerical values of the physical quantities like skin friction coefficient ($\tau )$ and local Nusselt number (*Nu)* are calculated for the diverse ranges of the parameters $M, \phi , Gr, Pr, K, R, Ec, t, a$ and $Q$. These physical quantities are calculated for both nanofluid particles, i.e. for $Cu$ and ${TiO}_{2}$ in Table 2.

**Table 2:** The values of $\tau$ and *Nu* for $Cu$ and ${TiO}_{2}$ in the numerical form.

$M$	$\phi$	$Gr$	$Pr$	$K$	$R$	$Ec$	$t$	$a$	$Q$	Cu		TiO_2_	
										$\tau$	$Nu$	$\tau$	$Nu$
1	0.001	2	6.2	0.5	2	0.02	0.1	0.5	2	2.2652	2.7903	2.2959	2.7905
2										2.3721	2.7899	2.4027	2.7901
3										2.476	2.7895	2.5063	2.7897
1	0.01									1.897	2.7698	2.1942	2.7719
	0.03									1.1491	2.7236	1.9786	2.7305
	0.001	3								2.164	2.7902	2.2127	2.7905
		4								2.0629	2.7901	2.1294	2.7904
		2	1							2.164	2.7902	2.2127	2.7905
			3							2.3662	2.7904	2.3791	2.7906
				1						2.154	2.7908	2.1854	2.791
				100						2.0405	2.7913	2.0726	2.7915
				0.5	3					2.078	2.7911	2.1098	2.7913
					4					2.0684	2.7912	2.1002	2.7914
					2	0.2				2.2451	2.2885	2.2794	2.2896
						0.5				2.2116	1.457	2.2519	1.4587
						0.02	0.3			2.5242	2.778	2.5545	2.7782
							0.5			2.8103	2.7629	2.8402	2.7631
							0.1	1		2.3914	2.7845	2.5545	2.7782
								1.5		2.5242	2.778	2.8402	2.7631
									0	2.2744	3.0894	2.3036	3.0897
									− 1	2.2788	3.2313	2.3072	3.2316

## Conclusions

Study the heat transmission characteristics of electrically conducting nanofluid flow by considering the solid volume fraction of nanoparticles $Cu$ and ${TiO}_{2}$ in the existence of viscous dissipation and radiation over a porous plate. The leading PDEs are tackled via the Galerkin weighted residual numerical approach. The influence of pertinent parameters is measured on the non-dimensional boundary layer distributions of velocity and temperature. Thus following concluding remarks can be depicted:

- The fluid velocity is efficiently controlled with a magnetic field and porous medium effects.
- The fluid velocity enhances with the rising level of radiation, Grashoff’s number, exponent coefficient, Eckert number, heat generation/absorption, and time.
- The fluid temperature is decreased during the heat absorption process.
- The consequence of the solid volume fraction, Eckert number, and heat generation is to escalate the fluid temperature.
- The heat transfer rate is not significantly affected by the magnetic field for $Cu$ and ${TiO}_{2}$.
- The thermal radiation and viscous dissipation decay the heat transfer rate.

Finally, further research can be considered by incorporating different nanoparticles in the fluid to study their thermal enhancement under a vertical plate for hybrid and ternary hybrid nanofluids. The G-FEM could be a potential utilization for future science and technology challenges^44–58^.

## Data Availability

All data generated or analyzed during this study are included in this published article.

## List of symbols

${{\varvec{u}}}^{*}$: The factor of nanofluid velocity in the path of ${\varvec{x}}$-axis $({\mathrm{ms}}^{-1})$.
$g$: Acceleration due to gravity $({\mathrm{ms}}^{-2})$
${\rho }_{f}$: Nanoparticles density (kg m^−3^)
${\rho }_{nf}$: Nanofluid density (kg m^−3^)
${{\varvec{T}}}^{*}$: Temperature of the nanofluid (K)
${\mu }_{nf}$: Viscosity of the nanofluid (kg m^−1^ s^−1^)
$$\sigma$$: Electrical conductivity
${\beta }_{nf}$: Volumetric coefficient of thermal expansion of the nanofluid
${k}_{nf}$: Nanofluid thermal conductivity (Wm^−1^ K)
${q}_{r}^{*}$: Radiation flux (Wm^−2^)
$K$: The permeability of medium
${\left({c}_{p}\right)}_{nf}$: Specific heat at constant pressure of the nanofluid
${\varvec{\nu}}$: The coefficient of kinematics viscosity
$$M$$: Magnetic parameter
$$Pr$$: Prandtl number
$$Gr$$: Grashoff number
${\varvec{R}}$: Thermal radiation factor
$Q$: Heat source/sink parameter
${{\varvec{K}}}_{{\varvec{p}}}$: The permeability parameter.
$$T$$: Time [s]
${\mu }_{f}$: Fluid viscosity (kg m^−1^ s^−1^)
$\phi$: Volume fraction (mol m^−3^)
